# Changes in Lipid Metabolism Enzymes in Rat Epididymal Fat after Chronic Central Leptin Infusion Are Related to Alterations in Inflammation and Insulin Signaling

**DOI:** 10.3390/ijms24087065

**Published:** 2023-04-11

**Authors:** María E. Casado, Sandra Canelles, Eduardo Arilla-Ferreiro, Laura M. Frago, Vicente Barrios

**Affiliations:** 1 Department of Endocrinology, Instituto de Investigación La Princesa, Hospital Infantil Universitario Niño Jesús, E-28009 Madrid, Spain; 2 Centro de Investigación Biomédica en Red de Fisiopatología de la Obesidad y Nutrición (CIBEROBN), Instituto de Salud Carlos III, E-28029 Madrid, Spain; 3 Department of Biological Systems, Faculty of Medicine, Universidad de Alcalá, E-28871 Alcala de Henares, Spain; 4 Department of Pediatrics, Faculty of Medicine, Universidad Autónoma de Madrid, E-28029 Madrid, Spain

**Keywords:** adipose tissue, cytokines, inflammation, insulin resistance, leptin, lipolysis

## Abstract

Leptin inhibits food intake and reduces the size of body fat depots, changing adipocyte sensitivity to insulin to restrain lipid accrual. This adipokine may modulate the production of cytokines that could diminish insulin sensitivity, particularly in visceral adipose tissue. To explore this possibility, we examined the effects of chronic central administration of leptin on the expression of key markers of lipid metabolism and its possible relationship with changes in inflammatory- and insulin-signaling pathways in epididymal adipose tissue. Circulating non-esterified fatty acids and pro- and anti-inflammatory cytokines were also measured. Fifteen male rats were divided into control (C), leptin (L, icv, 12 μg/day for 14 days), and pair-fed (PF) groups. We found a decrease in the activity of glucose-6-phosphate dehydrogenase and malic enzyme in the L group, with no changes in the expression of lipogenic enzymes. A reduction in the expression of lipoprotein lipase and carnitine palmitoyl-transferase-1A, together with a decrease in the phosphorylation of insulin-signaling targets and a low-grade inflammatory pattern, were detected in the epididymal fat of L rats. In conclusion, the decrease in insulin sensitivity and increased pro-inflammatory environment could regulate lipid metabolism, reducing epididymal fat stores in response to central leptin infusion.

## 1. Introduction

Adipose tissue is considered an endocrine organ, as it synthesizes numerous hormones, interleukins, and other factors that mediate different physiological processes at distant target tissues. Obesity generates an imbalance in this tissue, promoting changes in metabolism and insulin sensitivity, inflammation, and other related processes [1]. It is important to distinguish between subcutaneous and visceral adipose tissue actions. These tissues differ in their expression patterns of different factors, with partial or total removal of visceral fat being shown to improve insulin sensitivity in rodents [2], whereas transplantation of subcutaneous fat is reported to generate positive effects on metabolic processes [3].

In visceral adipose tissue, it is important to distinguish between retroperitoneal and epididymal fat, since they have different responses. Indeed, caloric restriction in mice differentially alters the expression of adipokines in these fat depots, with no modification of M2 macrophage markers in epididymal fat [4]. This type of gonadal fat is present only in males and is composed of several cell types, including mature adipocytes, immune cells, and endothelial cells. This tissue is involved in the synthesis and secretion of inflammatory factors in obese animals, with drugs used in the treatment of obesity mitigating reticulum stress, as well as the expression of inflammatory cytokines in this location [5].

Leptin is an adipokine secreted preferentially by adipose tissue, and, together with insulin, regulates energy homeostasis, improving systemic insulin sensitivity. However, to perform this exquisite modulation, it exerts different systemic actions depending on the location. In tissues such as muscle and liver, this adipokine has insulin-sensitizing actions and promotes the use of glucose, while it suppresses it in adipose tissue [6]. These insulin-related metabolic actions of leptin are mediated through insulin receptor substrate (IRS) phosphorylation and subsequent Akt activation [7]. In addition to reducing insulin-stimulated glucose metabolism in fat, leptin can modify lipid synthesis and the expression of enzymes associated with fatty acid oxidation in adipocytes [8]. It has also been shown that leptin administration reduces visceral fat content independently of food intake [9].

Obesity is usually characterized by the presence of high serum leptin levels in pro-portion to adiposity. In a situation of hyperleptinemia, increased cytokines can activate c-jun N-terminal kinase (JNK) and vice versa, both implicated in the generation of insulin resistance [10]. Leptin triggers signal transducers as well as the activator of transcription 3 (STAT3) and p38 mitogen-activated protein kinase (p38MAPK) phosphorylation, thus aggravating the inflammatory environment by enhancing synthesis of pro-inflammatory cytokines [11]. These events inhibit insulin signaling at several levels, such as IRS and phosphatidylinositol-3 kinase (PI3K) [12], and insulin resistance can also potentiate the synthesis of inflammatory interleukins in adipose cells [13].

Crosstalk between leptin and insulin signaling regulates not only intermediate metabolism, but also shared signaling targets. In this way, the effects of leptin on glucose metabolism, as well as on insulin signaling, are divergent in several organs and adipose tissue depots, increasing glucose metabolism and insulin signaling in the skeletal muscle and liver and reducing it in several fat depots [6,14]. Insulin signaling modulates fatty acid synthesis, increasing the generation of reduced nicotinamide adenine dinucleotide phosphate (NADPH) for lipid synthesis [15] and the expression of key enzymes for fatty acid synthesis through PI3K/Akt signaling [16].

Hormone-sensitive lipase (HSL) is the rate-limiting step for lipolysis of triglycerides (TGs) in fat cells [17], and lipoprotein lipase (LPL) regulates the hydrolysis of serum TGs, which delivers fatty acids to adipose sites. Usually, overweight and moderate obesity are accompanied by normal LPL activity, whereas insulin resistance conditions show a decrease in this hydrolytic activity [18]. The amount of visceral fat also depends on the oxidation of fatty acids through carnitine palmitoyl-transferase (CPT)1A, which controls mitochondrial uptake of fatty acids. In this way, high-fat diets generate insulin resistance and decrease the expression of genes associated with β-oxidation and mitochondrial biogenesis in epididymal fat [19].

We have previously reported that central leptin infusion increased some immune markers of inflammation and reduced phosphorylation of Akt in epididymal fat [20], a key marker of insulin signaling. Nevertheless, the activation of factors that regulate Akt activation and their relationship with changes in inflammatory pathways that modulate cytokine production were not explored in this model. For this reason, herein, we examined the effect of chronic exposure to increased leptin levels on the inflammatory environment, insulin-related signaling, and its relationship with the expression of key markers of lipid metabolism in the epididymal adipose tissue of male Wistar rats. To discriminate between the direct effects of leptin and effects due to reduced food intake, which can change insulin sensitivity and cytokine production [21], a group of pair-fed (PF) rats was included. Lastly, the possible contribution of epididymal fat to peripheral non-esterified fatty acid (NEFA) and cytokine levels was also evaluated.

## 2. Results

### 2.1. Characteristics of the Experimental Groups

We previously reported that food intake and body weight gain were decreased in the PF and L groups. Serum leptin was augmented in L, and insulin did not change after leptin treatment [22]. Visceral fat mass was reduced in both PF and L (*p* < 0.01), being lower (*p* < 0.01) in the L group (3.59 ± 0.21, 2.26 ± 0.14 and 1.72 ± 0.09 g in the C, PF, and L groups, respectively). The percentage of fat with respect to total body weight was diminished in PF and L rats compared to the controls (*p* < 0.05), although there were no differences between the PF and L groups (1.34 ± 0.07, 0.90 ± 0.05 and 0.77 ± 0.04 in the C, PF, and L groups, respectively).

### 2.2. Leptin Reduces Peripheral NEFAs and Triglycerides (TG)

Serum NEFA levels were reduced (*p* < 0.05) in the L group with respect to C and PF rats (17.52 ± 1.14, 20.94 ± 1.03 and 13.40 ± 1.35 mg/dL in C, PF and L groups, respectively), as were peripheral TG (*p* < 0.05) concentrations (36.82 ± 3.60, 32.51 ± 2.17 and 22.66 ± 2.38 mg/dL in the C, PF, and L groups, respectively).

### 2.3. Effect of Leptin Infusion on Lipid Anabolism-Related Parameters in Epididymal Fat

**Figure 1:**
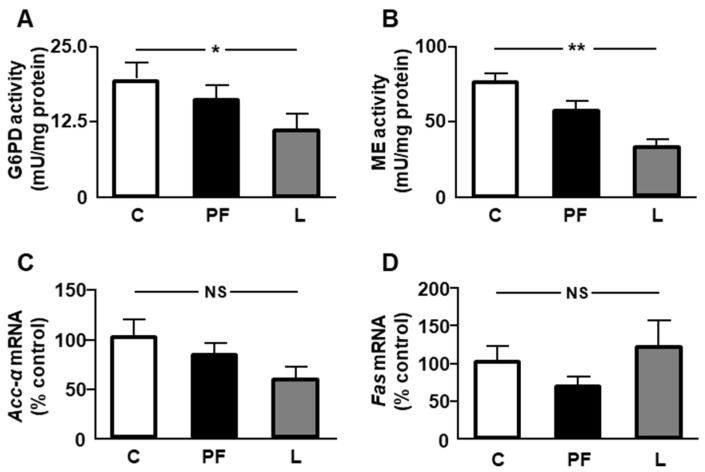
Effects of central leptin infusion on the activity/expression of markers of lipid anabolism in epididymal adipose tissue. Activity of (**A**) glucose 6 phosphate dehydrogenase (G6PD) and (**B**) malic enzyme (ME) and relative mRNA content of (**C**) acetyl CoA carboxylase (*Acc*)-*α* and (**D**) fatty acid synthase (*Fas*) in control rats (C), pair-fed rats (PF), and rats receiving a chronic icv leptin infusion (L). Data are presented as means ± SEM. *n* = 5. NS, non-significant. * *p* < 0.05, ** *p* < 0.01.

The activity of two enzymes that generate NADPH, necessary for lipid synthesis, were analyzed. A decrease in the activity of glucose-6-phosphate dehydrogenase (G6PD) and malic enzyme (ME) was detected in leptin-treated rats (Figure 1A,B, respectively). Relative mRNA levels of acetyl CoA carboxylase (*Acc*)-*α* and fatty acid synthase (*Fas*) showed no differences among the experimental groups (Figure 1C,D, respectively).

### 2.4. Leptin Infusion Reduces the Expression of PPAR-γ and Lipid Metabolic Markers in Epididymal Fat

**Figure 2:**
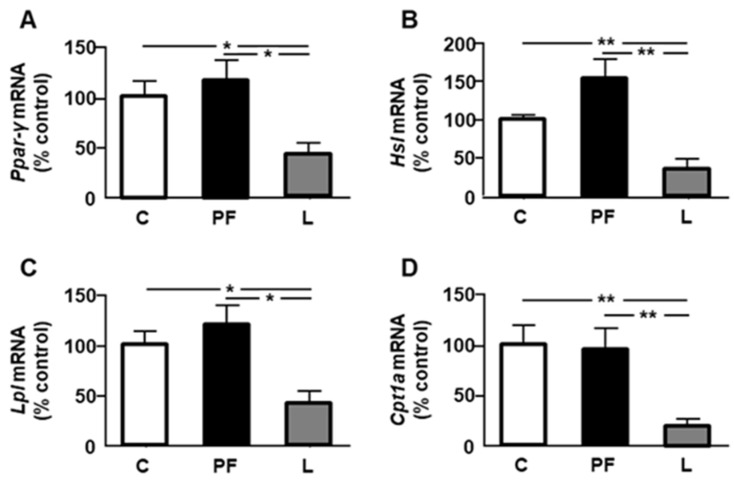
Effects of central leptin on the relative mRNA content of (**A**) peroxisome proliferator-activated receptor *(Ppar)-γ*, (**B**) hormone-sensitive lipase (*Hsl*), (**C**) lipoprotein lipase (*Lpl*), and (**D**) carnitine palmitoyl-transferase *(Cpt)1a* in the epididymal fat of control rats (C), pair-fed rats (PF), and rats receiving a chronic icv leptin infusion (L). Data are presented as means ± SEM. *n* = 5. * *p* < 0.05, ** *p* < 0.01.

We next studied selected parameters related to insulin sensitivity and lipid catabolism. Relative mRNA levels of peroxisome proliferator-activated receptor *(Ppar)-γ* (Figure 2A), *Hsl* (Figure 2B), *Lpl* (Figure 2C), and *Cpt1a* (Figure 2D) were decreased in leptin-treated rats, with no effect of pair feeding on any of these parameters.

### 2.5. Chronic Leptin Increases Activation of Inflammatory Signaling Targets in Epididymal Fat

**Figure 3:**
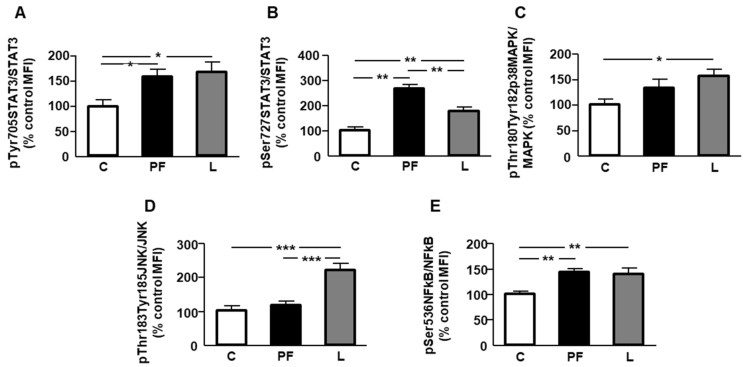
Effect of chronic central leptin treatment on the activation of inflammatory signaling targets in epididymal adipose tissue. Relative protein levels of (**A**) signal transducer and activator of transcription 3 (STAT3) phosphorylated (p) at Tyr705 (pTyr705STAT3), (**B**) STAT3 phosphorylated at Ser727 (pSer727STAT3), (**C**) p38 mitogen-activated protein kinase (MAPK) phosphorylated at Thr180 and Tyr182 (pThr180Tyr182p38MAPK), (**D**) c-jun-N-terminal kinase (JNK) phosphorylated at Thr183/Tyr185 (pThr183/Tyr185JNK), and (**E**) nuclear factor kappa B (NFkB) phosphorylated at Ser536 (pSer536NFkB) in control rats (C), pair-fed rats (PF), and rats receiving a chronic icv leptin infusion (L). Data are presented as means ± SEM. *n* = 5. * *p* < 0.05, ** *p* < 0.01, *** *p* < 0.001.

Phosphorylation of signal transducer and activator of transcription (STAT)3 in tyrosine 705 was increased in PF and L groups, without differences between them (Figure 3A), and phosphorylation of STAT3 in serine 727 residue was also augmented in both PF and L rats, being higher in the PF group (Figure 3B). Phosphorylation of p38MAPK was increased in leptin-treated rats (Figure 3C), and activation of JNK was increased in the L group compared to C and PF rats (Figure 3D). Finally, phosphorylation of nuclear factor κB (NFκB) was augmented in both PF and L rats, with no differences between these groups (Figure 3E).

### 2.6. Leptin Reduced Insulin-Related Signaling in Epididymal Fat

**Figure 4:**
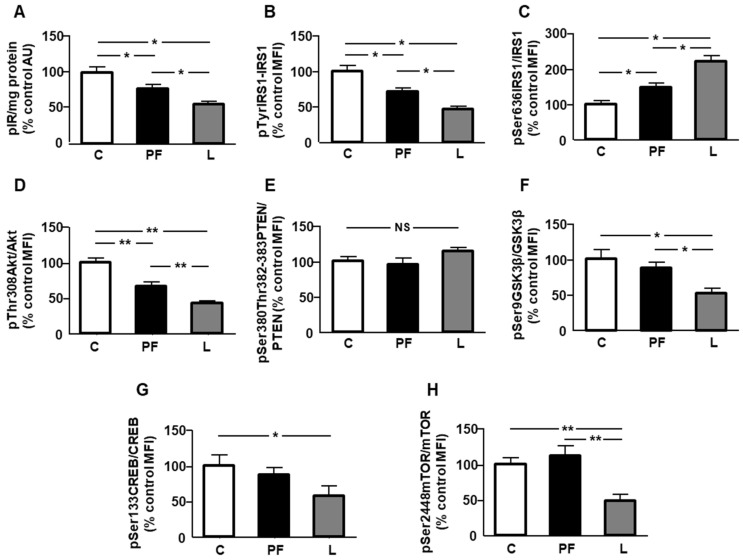
Effects of central leptin infusion on insulin-related signaling in epididymal adipose tissue. Relative protein levels of (**A**) phosphorylated (p) insulin receptor (pIR), (**B**) insulin receptor substrate (IRS)1 phosphorylated at Tyr residues (pTyrIRS1), (**C**) IRS1 phosphorylated at Ser636 (pSer636IRS1), (**D**) Akt phosphorylated at Thr308 (pThr308Akt), (**E**) phosphatase and tensin homolog (PTEN) phosphorylated at Ser380 and Thr382-383 (pSer380Thr382-383PTEN), (**F**) glycogen synthase kinase (GSK)3β at Ser9 (pSer9GSK3β), (**G**) cAMP-response element binding protein (CREB) at Ser133 (pSer133CREB), and (**H**) mechanistic target of rapamycin (mTOR) phosphorylated at Ser2448 (pSer2448mTOR) in control rats (C), pair-fed rats (PF), and rats receiving a chronic icv leptin infusion (L). Data are presented as means ± SEM. *n* = 5. AU, absorbance units; MFI, median fluorescent intensity; NS, non-significant. * *p* < 0.05, ** *p* < 0.01.

Phosphorylation of insulin receptor (IR) and IRS1 on tyrosine residues was reduced in PF and L rats, being lower in the L group (Figure 4A,B, respectively). Phosphorylation of IRS1 on serine residues is known to reduce insulin signaling [23]. We found an increase in both the PF and L groups, being higher in leptin-treated rats (Figure 4C). Phosphorylation of Akt in threonine was reduced in both the PF and L groups, with this decrease being less significant in the L group (Figure 4D). Chronic central leptin infusion or pair feeding did not modify phosphorylation of phosphatase and tensin homolog deleted on chromosome 10 (PTEN, Figure 4E). Phosphorylation of glycogen synthase kinase (GSK)3β was reduced in leptin-treated rats (Figure 4F), and phosphorylation of cyclic AMP-response element binding protein (CREB) was decreased in L rats (Figure 4G). Finally, phosphorylation of mechanistic target of rapamycin (mTOR) in Ser2448 (pSer2448mTOR) was reduced in leptin-treated rats, with respect to the C and PF groups (Figure 4H).

### 2.7. Changes in Inflammatory Markers after Leptin Infusion

**Figure 5:**
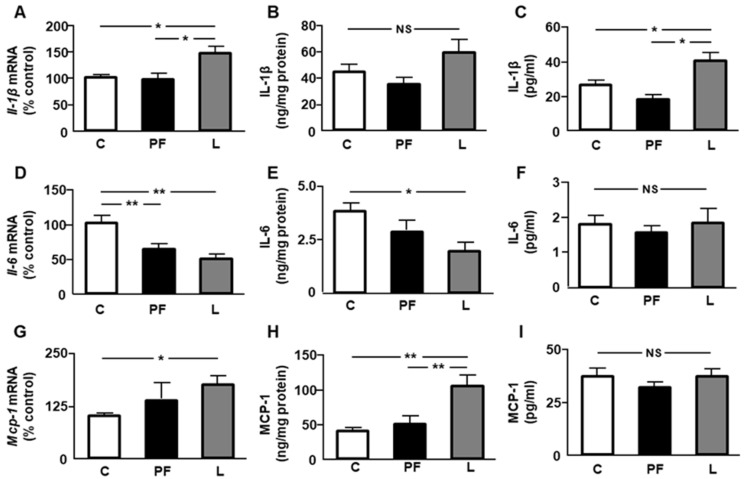
Effect of chronic central leptin treatment on the relative mRNA content of (**A**) interleukin *(Il)-1β*, (**D**) *Il-6,* and (**G**) monocyte chemoattractant protein *(Mcp)-1*; the protein concentrations of (**B**) IL-1β, (**E**) IL-6, and (**H**) MCP-1 in epididymal fat; and the serum concentrations of (**C**) IL-1β, (**F**) IL-6, and (**I**) MCP-1 in control rats (C), pair-fed rats (PF), and rats receiving a chronic icv leptin infusion (L). Data are presented as means ± SEM. *n* = 5. NS, non-significant. * *p* < 0.05, ** *p* < 0.01.

Relative mRNA levels of interleukin *(Il)-1β* were higher in the epididymal fat of L compared to both C and PF rats (Figure 5A), whereas its protein concentrations remained unchanged (Figure 5B). Serum IL-1β levels were augmented in leptin-treated rats (Figure 5C). Epididymal fat mRNA levels of *Il-6* were decreased in PF and L rats, with no differences between them (Figure 5D). Visceral IL-6 protein content was reduced only in L rats (Figure 5E), and serum IL-6 levels remained unchanged (Figure 5F). Monocyte chemoattractant protein *(Mcp)-1* mRNA levels were augmented in L compared to C rats (Figure 5G), and its visceral protein concentrations were increased in L with respect to the C and PF groups (Figure 5H). Circulating MCP-1 concentrations presented no differences among the experimental groups (Figure 5I).

### 2.8. Leptin Induces Changes in Anti-Inflammatory Interleukins

**Figure 6:**
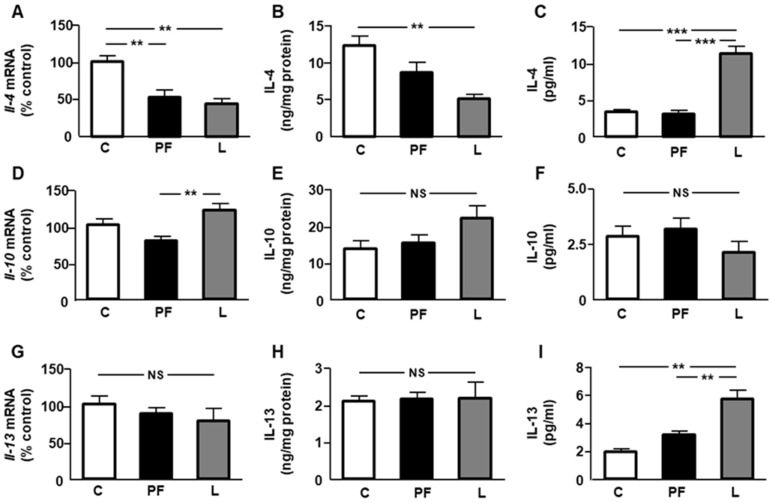
Effect of central leptin infusion on the relative mRNA content of (**A**) interleukin *(Il)-4*, (**D**) *Il-10,* and (**G**) *Il-13*; the protein concentrations of (**B**) IL-4, (**E**) IL-10, and (**H**) IL-13 in epididymal fat; and the serum concentrations of (**C**) IL-4, (**F**) IL-10, and (**I**) IL-13 in control rats (C), pair-fed rats (PF), and rats receiving a chronic icv leptin infusion (L). Data are presented as means ± SEM. *n* = 5. NS, non-significant. ** *p* < 0.01, *** *p* < 0.001.

When we studied the epididymal fat mRNA levels of *Il-4*, a reduction in PF and L rats (Figure 6A) was found, while IL-4 protein content was reduced only in leptin-treated rats (Figure 6B). Circulating IL-4 levels were increased in L rats compared to the other groups (Figure 6C). An increase in *Il-10* mRNA levels was observed in L compared to PF rats, but no differences in its level in fat or serum were found (Figure 6B,C, respectively). No differences in *Il-13* mRNA levels or protein levels were detected among the experimental groups (Figure 6G,H, respectively). Lastly, serum IL-13 concentrations were augmented in L with respect to the C and PF groups (Figure 6I).

### 2.9. Correlation of Inflammatory Targets and Markers of Insulin Sensitivity with the Expression of Enzymes of Lipid Metabolism

Leptin is linked to the regulation of several inflammatory and insulin-sensitizing markers that modulate lipid metabolism [24]. Linear regression analyses showed negative relationships of some inflammatory targets and enzymes of lipid catabolism and positive correlations of these enzymes with insulin-sensitizing markers (Table 1). 

## 3. Discussion

Dysfunctional visceral fat is associated with the initiation and maintenance of inflammation and, later, insulin resistance, which can occur with or without obesity [25]. Herein, we report a reduction in the activity of key enzymes of the pentose phosphate cycle, as well as in the expression of enzymes of fatty acid catabolism in the epididymal fat of rats after central leptin infusion. These changes are accompanied by a slight increase in inflammation and the generation of resistance to the action of insulin in this localization. Most of the variations in the cytokine profile and insulin signaling were not observed in pair-fed rats, suggesting an effect of leptin that is not related to the decrease in food intake. Finally, we provide evidence that modifications in the expression levels of pro- and anti-inflammatory cytokines in this fat depot did not significantly contribute to their circulating levels, whereas the reduction in fat lipolytic activity may have contributed to decreased NEFA levels in leptin-treated rats.

The reduction in epididymal fat mass after leptin infusion which we observed in this study has been previously reported [26], and may be related to the effect of leptin on the suppression of glucose metabolism and modification of insulin sensitivity [6]. Some compounds involved in lipid signaling induce concomitant weight loss and visceral fat reduction, while also increasing inflammation in diet-induced obesity [27]. The decrease in epididymal fat mass could be related with a reduction in lipid anabolism. Indeed, we found a reduction in G6PD and ME activity, crucial enzymes for the generation of NADPH, a cofactor required for fatty acid synthesis [28].

We have previously found that phosphorylation of Akt in Ser473 was reduced in epididymal fat after chronic leptin infusion [20]. In this study, we demonstrated that the phosphorylation of Akt in Thr308 was also diminished. This is an interesting finding, because phosphorylation at both sites is needed for the transduction of activation signals of the kinase [29]. Reduction in the generation of NADPH for fatty acid synthesis may be also associated with decreased Akt activation in Thr308 and Ser473, as Akt phosphorylates NAD kinase [30]. In addition, the reduction in the production of NADPH could be directly associated with the loss of visceral adipose tissue, since deficient synthesis under nutrient stress conditions causes inflammation and cell death [31]. The decrease in Akt phosphorylation may affect lipid synthesis through down-regulation of CREB. In fact, there is a close relationship between Akt and CREB phosphorylation [32], and activation of this factor can upregulate the expression of several enzymes involved in fatty acid synthesis, including ACC-α, as well as the content of triacylglycerol [33]. In addition, more compelling evidence for CREB in the control of lipid anabolism was obtained via CREB knockdown with antisense oligonucleotides. Findings verified that it not only diminished the content of TG in several organs, but also altered the expression of key genes involved in fatty acid synthesis [34].

A decrease in ME activity can affect the synthesis of lipogenic enzymes, as it has been reported that transgenic mice that overexpress this enzyme present an induction of lipogenic pathway genes [35]. Although we found no changes in the expression of enzymes involved in fatty acid synthesis, a non-significant reduction in mRNA levels of acetyl CoA carboxylase (around 40%) indicates that synthesis could be affected by leptin infusion. This apparent reduction could be related to reduced phosphorylation of mTOR, as mTOR activation increases proliferation and lipid anabolism [36]. Regarding the mechanism involved, it is known that its activation regulates lipid metabolism by phosphorylation of lipin-1, which allows nuclear sterol regulatory element binding protein-1c to bind to lipogenic genes, causing the promotion of lipogenesis [37]. The decline in the expression of LPL can also affect this fat depot, since this enzyme hydrolyzes fatty acids from the blood for their subsequent incorporation into different tissues. However, other enzymes could counteract the decrease in epididymal adipose tissue in leptin-infused rats. The decrease in HSL mRNA levels limits the lipolysis of TG in adipocytes, and subsequent liberation as well as the reduced CPT1A expression could restrict the oxidation of free fatty acids in the mitochondria.

Additional factors could be implicated in the changes in lipid metabolism observed in leptin-treated rats. In this way, leptin treatment in adipocytes induces a pro-inflammatory cytokine pattern, modulating the synthesis of TNF-α, IL-10, and IL-6 [38], among other factors. Leptin can trigger mediators of inflammation such as STAT3, p38MAPK, and JNK [39], whereas the deficiency of leptin-induced STAT3 signaling is associated with reduced inflammation [40]. Usually, adipose tissue macrophages exhibit an anti-inflammatory profile, but in presence of high leptin levels, a polarization to a pro-inflammatory phenotype takes place [41] through activation of STAT3 and JNK, causing macrophages to interact with adipocytes in order to promote the synthesis of pro-inflammatory interleukins [42]. The increase in IL-1β expression observed in this study could enhance the inflammatory response, attracting inflammatory cells to epididymal fat, whereas a reduction in local IL-4 levels could be related to decreased insulin sensitivity [43].

Direct actions of leptin on cell metabolism are rather unusual, and are mainly mediated by its effects on insulin sensitivity in adipocytes [8]. We found a reduction in the phosphorylation of the insulin receptor and downstream targets of this pathway in the epididymal fat of pair-fed and leptin-infused rats, which was more significant in the latter group, and this inhibited insulin’s actions in adipocytes [44]. In addition, JNK activation in leptin-infused rats could interfere with insulin signaling by increasing IRS1 degradation and triggering IRS1 serine phosphorylation in fat, thus lessening the signaling and promoting inflammation [45].

An increase in Akt activation inactivates GSK3β through phosphorylation at serine 9 residue [46]. We found a decrease in GSK3β phosphorylation, likely due to reduced Akt phosphorylation, which is compatible with the low-grade inflammatory profile detected in this study. GSK3β is implicated in inflammatory processes, increasing IL-1β and MCP-1, among other pro-inflammatory cytokines, in the adipose tissue of women with gestational diabetes [47]. Additionally, it has been demonstrated that GSK3β increases NFκB activation, whereas its inhibition reduces NFκB signaling and inflammation [48].

We found a reduction in the expression of peroxisome proliferator-activated receptor (PPAR)-γ in leptin-treated rats. PPAR-γ belongs to a family of nuclear receptors that can function as lipid sensors and is a receptor for insulin-sensitizing drugs, with its expression being activated by insulin and nutrition and downregulated by fasting, insulin-deficient diabetes, and inflammation [49]. An inverse relationship between the increase in IL-1β and MCP-1, among other pro-inflammatory cytokines, and the expression of PPAR-γ in adipocytes has been reported [50], similar to that found in this study. PPAR-γ signaling plays an important role in the regulation of fatty acid metabolism, modulating the expression of genes involved in the carnitine acyltransferase pathway. Decreased PPAR-γ mRNA levels correlate with the disruption of carnitine homeostasis [51]; moreover, small interfering RNA-mediated PPAR-γ knockdown reduced CPT1 and CPT2 [52].

It has been known for more than two decades that the administration of leptin to obese mice decreases LPL activity in epididymal fat [53]. Activation of PPAR-γ is associated with preferential LPL-mediated fatty acid storage in adipose tissue through transcriptional control of this lipase [54], whereas some abnormalities in lipoprotein metabolism that cause insulin resistance decrease both PPAR-γ and LPL [55]. In addition, a pro-inflammatory environment could play an important role in reducing not only the expression, but also the enzymatic activity, of this lipase [56]. The increase in IL-1β and other interleukins reduces transcription, as well as specific inhibitors for the PI3K/AKT signaling pathway [57].

Our findings showed a reduction in HSL expression in epididymal adipose tissue, together with a diminution in circulating NEFA and TG levels after leptin administration. In this regard, it has been reported that the contribution of visceral adipose tissue to circulating levels of NEFA and TG is greater than that of subcutaneous tissue [58]. Thus, the decrease in the expression of HSL in epididymal fat observed herein in leptin-treated rats may contribute to a lower release of lipids into the bloodstream, as we detected in this experimental group.

A surprising result was the decrease in the expression of HSL, along with a reduction in the activation of the insulin-signaling pathway, since insulin exerts a suppressive effect on this lipase via central and peripheral systems [59]. However, it has been shown that the suppressive action of insulin occurs when adipocyte cultures are exposed to insulin in the presence of high glucose levels [60], which we have not previously reported in this experimental model [61]. Furthermore, some flavonoids that reduce IL-1β and MCP-1 concentrations in adipose tissue increase those of HSL [62]. Thus, it could be that the high levels of IL-1β and MCP-1 found in this study affect the expression of this lipase. We found a reduction in IL-4 levels in the epididymal fat of leptin-treated rats that could affect mRNA levels of HSL, as IL-4 promotes lipolysis to decrease lipid deposits by enhancing HSL activity [63]. Finally, a reduction in CREB activation could affect HSL mRNA levels, as phosphorylation of this transcription factor increases HSL expression in adipocytes [64].

Several aspects should be considered when evaluating these results. The number of animals in each experimental group was low. Although we utilized a new, single set of animals, we have tested several parameters using old and current samples in the same three experimental groups with similar results. In addition, as we reported in previous studies performed using this experimental model with other tissues [14,65,66], we used five animals per group, estimating a 95% confidence level and reasonable and significant differences in the studied variables. In spite of the treatment performed, our results, for all studied parameters, showed low variability within each experimental group. Visceral adipose tissue consists of different cell types, and herein, we did not determine the contribution of adipocytes or other cell types, such as macrophages or monocytes, in the generation of an inflammatory response in this fat depot. In this way, concerning the effects on genes expressed by the stromal vascular fraction, it would be interesting to characterize the variation in the ratio of adipocytes to the stromal vascular fraction. Nevertheless, it is known that leptin administration decreases epididymal tissue mass [9], reducing the proliferation of both adipocyte and stromal fractions [67]. Therefore, we could speculate that changes in a similar proportion of visceral adipose cells and a reduction in epididymal fat after leptin infusion could partially counteract the pro-inflammatory effects of this adipokine, as observed in their circulating levels, which showed a mild inflammatory profile. Another caveat is that we determined the expression of key enzymes of lipid metabolism, but not their content or activity, in epididymal fat. These additional determinations could provide new information regarding its possible contribution to circulating levels of NEFAs and TGs.

In summary, our findings show that chronic exposure to increased leptin levels causes modifications in the physiological signals controlling lipid homeostasis in visceral fat. The mechanisms of regulation of lipid metabolism in this adipose tissue provoked by hyperleptinemia in this experimental model may be due to changes in its signaling effectors, as well as in crosstalk with insulin- and inflammatory-signaling pathways. In fact, leptin is a pleiotropic hormone that exerts numerous actions, most of them associated with the control of energy homeostasis and metabolism, these functions being modulated through the interaction of this adipokine with other hormones and cytokines. The prevention of these pathological changes in metabolism, as well as in low-grade inflammation induced by high leptin concentrations, must be investigated. Reasonable manipulation of leptin could be a possibility in therapy for metabolic diseases where hyperleptinemia is present. Efforts should also focus on the role of insulin-sensitizing and anti-inflammatory agents that can modify altered signaling pathways and reverse the abnormal lipid metabolism caused by elevated levels of this adipokine.

## 4. Materials and Methods

### 4.1. Materials

All chemicals were purchased from Merck (Darmstadt, Germany) unless otherwise noted. The high-capacity cDNA kit and TaqMan gene expression assays were acquired from Thermo Fisher Scientific (Waltham, MA, USA). The kit for RNA extraction was obtained from Qiagen (Hilden, Germany), and the high-capacity cDNA kit was purchased from Nzytech (Lisbon, Portugal).

### 4.2. Animals Treatment and Experimental Design

All procedures were carried out in accordance with the local ethics committee and complied with Royal Decree 53/2013 pertaining to the protection of experimental animals, as well as with the European Communities Council Directive (2010/63/EU). This study was approved by the Ethical Committee of Animal Experimentation of the Universidad de Alcalá (PROEX018/16, 14 June 2016). Male Wistar rats (250 ± 10 g) purchased from Harlan Laboratories (Barcelona, Spain) were individually caged and fed standard chow and water ad libitum. Animals were anesthetized using 4 mg of ketamine/100 g body weight (bw) and 0.5 mg of xylazine/100 g bw throughout the surgical procedures.

Fifteen rats were anesthetized, positioned in a stereotaxic apparatus, and treated as previously reported [22] after a fasting period of 12 h. Briefly, a cannula attached to an osmotic minipump (Alzet, Durect Corporation, Cupertino, CA, USA) with either saline or leptin (Preprotech, Rocky Hill, NJ, USA; delivering 12 μg/day at a volume of 0.5 µL/h) was implanted and conserved for two weeks. To differentiate the inhibitory effects of leptin on appetite, a pair-fed group that received the same amount of food as that ingested by the leptin-treated group the day prior was included. On the last day, after a fasting period of 12 h, rats were sacrificed. This resulted in the following groups (*n* = 5 per group): chronic vehicle (control, C), pair-fed with chronic vehicle (pair-fed, PF), and chronic leptin (leptin, L). Epididymal fat was isolated, and blood was collected. After 30 min at room temperature, the blood was centrifuged at 1500× *g* for 10 min at 4 °C and the serum was collected and frozen at −80 °C.

### 4.3. Tissue Homogenization and Protein Quantification

For the immunodetection of phosphorylated (p) Thr308Akt, Akt, pSer133CREB, CREB, pSer9GSK3β, GSK3β, IL-1β, IL-4, IL-6, IL-10, IL-13, pIR, pSer636-IRS1, pTyr-IRS1, IRS1, pThr183/Tyr185-JNK, JNK, pThr180/Tyr182-p38MAPK, p38MAPK, MCP-1, pSer2448mTOR, mTOR, pSer536-NFkB, NFkB, pSer727STAT3, pTyr705STAT3, and STAT3, epididymal fat was homogenized on ice in 400 µL of lysis buffer (Merck Millipore). The lysates were frozen for 12 h at −80 °C, then centrifuged at 12,000× *g* for 5 min at 4 °C. Supernatants were stored at −80 °C until assayed. Protein levels were determined by the Bradford method (Bio-Rad Laboratories, Madrid, Spain).

### 4.4. Serum NEFAs and TGs Levels

Concentrations were determined by using commercially available kits (Wako Chemicals, Neuss, Germany, and Spinreact, St. Esteve de Bas, Spain) following the manufacturer’s instructions. The average coefficients of variation for both kits were lower than 10%.

### 4.5. ELISA

The phosphorylation of insulin receptors was measured using an ELISA obtained from Assay Solution (Woburn, MA, USA) to detect phosphorylated insulin receptor β protein. After incubation with lysates of visceral fat, insulin receptor-β protein was bound by a monoclonal antibody. After washing, a detection antibody coupled with biotin was added. After incubation, a streptavidin–horseradish peroxidase complex was supplemented, and after washing, tetramethylbenzidine was used and the absorbance at 450 nm was measured. Intra- and inter-assay variation coefficients were lower than 10%.

### 4.6. Multiplexed Bead Immunoassay

Serum and tissue concentrations of the IL-1β, IL-4, IL-6, IL-10, IL-13, and MCP-1 proteins were phosphorylated, and total levels of Akt, CREB, GSK3β, IRS1, JNK, MAPK, mTOR, NFkB, and STAT3 in epididymal fat were measured using multiplexed bead immunoassay (MBIA) kits from Merck (Darmstadt, Germany) as previously described [68], with minor modifications [69]. Homogenized samples were incubated for 18 h at 4 °C with beads containing fluorescent labels and conjugated to the appropriate antibody for each parameter. After washing, wells were incubated for 30 min with the corresponding monoclonal antibodies coupled with biotin. Afterwards, beads were incubated for an additional period of 30 min, with streptavidin conjugated to phycoerythrin. After washing, beads were resuspended and a minimum of 50 beads/parameter were analyzed in the Bio-Plex suspension array system 200 (Bio-Rad Laboratories, Madrid, Spain). Raw data (mean fluorescence intensity) were analyzed using the Bio-Plex Manager Software 6.2. (Bio-Rad Laboratories).

### 4.7. Enzyme Activity Assays

#### 4.7.1. Glucose-6-Phosphate Dehydrogenase

The activity of G6PD [EC 1.1.1.49] was determined with a kit from Sigma-Aldrich following the manufacturer’s recommendations. Concisely, after homogenization of 20 mg of fat in phosphate buffer saline and subsequent centrifugation, diluted supernatants were incubated at 37 °C with master reaction mix, and absorbance was measured at 450 nm.

#### 4.7.2. Malic Enzyme

The activity of this enzyme (EC 1.1.1.40) was determined according to the method of Geer et al. [70]. Diluted supernatants were incubated at 25 °C with a triethanolamine buffer, malic acid, and NADP, and absorbance was checked at 340 nm. All chemicals were obtained from Sigma-Aldrich, St. Louis, MO, USA.

### 4.8. RNA Purification and Real Time PCR Analysis

Total RNA was extracted from the epididymal fat with the RNeasy Lipid Tissue Mini Kit (Qiagen, Hilden, Germany), and reverse transcription was carried out using the NZY First-Strand cDNA Synthesis Kit (NZYTech, Lisbon, Portugal). Real-time PCR was performed in an ABI Prism 7000 Sequence Detection System (Applied Biosystems, Carlsbad, CA, USA) using NZY qPCR Probe Master mix and the thermocycler parameters recommended by the manufacturer. The TaqMan gene expression assay was used for *Acc-α*, *Cpt1a*, *Fas*, *Hsl*, *Il-1β*, *Il-4*, *Il-6*, *Il-10*, *Il-13*, *Lpl*, *Mcp-1,* and *Ppar-γ* (Rn00573474_m1, Rn00580702_m1, Rn00569117_m1, Rn00563444_m1, Rn00580432_m1, Rn01456866_m1, Rn01410330_m1, Rn00563409_m1, Rn00587615_m1, Rn00561482_m1, Rn01453994_m1, and Rn00440945_m1, respectively). A relative gene expression comparison was carried out using an invariant endogenous control (actin). The ΔΔCT method was used for relative quantification.

### 4.9. Statistical Analysis

Data are represented as mean ± standard error of the mean (SEM). The analysis of all data was carried out by one-way ANOVA, followed by Bonferroni’s post hoc tests, using Statview (Statview 5.01, SAS Institute, Cary, NC, USA) software. Linear regression analysis was used to determine the relationships between specific parameters. Values were considered significantly different when the *p* value was less than 0.05.

## Figures and Tables

**Table 1:** Correlations between inflammatory-related targets and markers of insulin sensitivity with relative mRNA levels of enzymes of lipid metabolism.

	p-p38MAPK	p-JNK	MCP-1	mRNA *Ppar-γ*	p-IR	pTyr-IRS1	p-Akt
	*r p*	*r p*	*r p*	*r p*	*r p*	*r p*	*r p*
mRNA Acc-α	−0.26 NS	+0.29 NS	+0.31 NS	−0.36 NS	+0.37 NS	+0.33 NS	+0.36 NS
mRNA Fas	+0.14 NS	+0.22 NS	+0.37 NS	−0.30 NS	+0.21 NS	−0.08 NS	+0.10 NS
mRNA Hsl	−0.51 *	−0.65 *	−0.58 *	+0.84 ***	+0.42 NS	+0.38 NS	+0.39 NS
mRNA Lpl	−0.47 *	−0.71 **	−0.63 **	+0.99 ***	+0.48 *	+0.48 *	+0.45 *
mRNA Cpt1a	−0.35 NS	−0.60 **	−0.72 **	+0.87 ***	+0.61 **	+0.53 *	+0.47 *

*ACC-α*, acetyl CoA carboxylase α; p-Akt, phosphorylated (p) protein kinase B in serine 473; *Fas*, fatty acid synthase; *Cpt1a*, carnitine palmitoyl-transferase 1A; *Hsl*, hormone-sensitive lipase; p-JNK, phosphorylated c-Jun N-terminal kinase; p-IR, phosphorylated insulin receptor; pTyr-IRS-1, phosphorylated IR substrate 1 in tyrosine residues; *Lpl*, lipoprotein lipase; MCP-1, monocyte chemoattractant protein-1; *Ppar-γ*, peroxisome proliferator-activated receptor-γ, p-p38MAPK, phosphorylated p38 mitogen-activated protein kinase. Correlation coefficients (*r*) and *p* values are provided for all analyses. NS, non-significant. * *p* < 0.05, ** *p* < 0.01, *** *p* < 0.001.

## Data Availability

All relevant data are included within the manuscript.

## Abbreviations

ACC-α	Acetyl CoA carboxylase-α
Akt	Protein kinase B
CPT1A	Carnitine palmitoyl-transferase 1A
CREB	cAMP-response element binding protein
FAS	Fatty acid synthase
GSK3β	Glycogen synthase kinase 3β
G6PD	Glucose-6-phosphate dehydrogenase
HSL	Hormone-sensitive lipase
icv	Intracerebroventricular
IL	Interleukin
IR	Insulin receptor
IRS1	Insulin receptor substrate 1
JNK	c-Jun N-terminal kinase
LPL	Lipoprotein lipase
MCP-1	Monocyte chemoattractant protein-1
ME	Malic enzyme
mTOR	Mechanistic target of rapamycin
NADPH	Reduced nicotinamide adenine dinucleotide phosphate
NFkB	Nuclear factor kappa B
PF	Pair-fed
PPARγ	Peroxisome proliferator-activated receptor-γ
PTEN	Phosphatase and tensin homolog homolog deleted on chromosome 10
p38MAPK	p38 mitogen-activated protein kinase
STAT3	Signal transducer and activator of transcription 3
